# Genetic variability in a frozen batch of MCF-7 cells invisible in routine authentication affecting cell function

**DOI:** 10.1038/srep28994

**Published:** 2016-07-26

**Authors:** Andre Kleensang, Marguerite M. Vantangoli, Shelly Odwin-DaCosta, Melvin E. Andersen, Kim Boekelheide, Mounir Bouhifd, Albert J. Fornace, Heng-Hong Li, Carolina B. Livi, Samantha Madnick, Alexandra Maertens, Michael Rosenberg, James D. Yager, Liang Zhaog, Thomas Hartung

**Affiliations:** 1 Center for Alternatives to Animal Testing (CAAT), Department of Environmental Health Sciences, Bloomberg School of Public Health, Johns Hopkins University Baltimore, MD, USA; 2 Department of Pathology and Laboratory Medicine, Brown University, Providence, RI, USA; 3 The Hamner Institutes for Health Sciences, Research Triangle Park, NC, USA; 4 Department of Biochemistry and Molecular & Cellular Biology, and Lombardi Comprehensive Cancer Center, Georgetown University, Washington, DC, USA; 5 Agilent Technologies, Inc., Santa Clara, CA, USA; 6 Department of Environmental Health Sciences, Bloomberg School of Public Health, Johns Hopkins University, Baltimore, MD, USA; 7 University of Konstanz, CAAT-Europe, Germany

## Abstract

Common recommendations for cell line authentication, annotation and quality control fall short addressing genetic heterogeneity. Within the Human Toxome Project, we demonstrate that there can be marked cellular and phenotypic heterogeneity in a single batch of the human breast adenocarcinoma cell line MCF-7 obtained directly from a cell bank that are invisible with the usual cell authentication by short tandem repeat (STR) markers. STR profiling just fulfills the purpose of authentication testing, which is to detect significant cross-contamination and cell line misidentification. Heterogeneity needs to be examined using additional methods. This heterogeneity can have serious consequences for reproducibility of experiments as shown by morphology, estrogenic growth dose-response, whole genome gene expression and untargeted mass-spectroscopy metabolomics for MCF-7 cells. Using Comparative Genomic Hybridization (CGH), differences were traced back to genetic heterogeneity already in the cells from the original frozen vials from the same ATCC lot, however, STR markers did not differ from ATCC reference for any sample. These findings underscore the need for additional quality assurance in Good Cell Culture Practice and cell characterization, especially using other methods such as CGH to reveal possible genomic heterogeneity and genetic drifts within cell lines.

Recently, there has been a call for increased attention to cell line authentication, annotation and quality control, which, if not carefully documented and described, can seriously affect reproducibility and scientific quality^1^,^2^,^3^. Since much of what we know about the molecular mechanisms of cancer is derived from these cell lines, and they are broadly used for drug development and regulatory testing, this represents a key concern for putting such investigations on a sound footing.

The human breast adenocarcinoma cell line MCF-7 (Michigan Cancer Foundation-7) has served for over 40 years as a standard model for *in vitro* cancer research as well as estrogen and progesterone receptor science^4^,^5^ and is one of the key cancer cell lines used as a model for investigation of processes that impact patient care^6^. Almost 23,000 articles using MCF-7 can be retrieved in PubMed; it is used for both basic and applied sciences such as oncologic mechanisms, characterization of drug effects, as well as endocrine disruption hazard assessment of chemicals. However, it is not clear whether all studies of MCF-7 cells actually use the same entity. As early as 1987, Resnicoff *et al.* identified subpopulations in MCF-7 by Percoll gradient centrifugation that showed differences in growth rate, DNA synthesis and expression of estrogen receptors and pointed out the heterogeneous character of MCF-7^7^. Later, these findings were confirmed by others^8^,^9^,^10^ and it is now recognized that MCF-7 is heterogeneous with respect to both the expression of hormone receptors and to the utilization of the signaling pathways linked to these receptors, differences that result in phenotypic heterogeneity^11^. Sub-clones vary in estrogen and progesterone receptor expression, as well as epidermal growth factor. However, genotyping analysis shows that all sub-clones are related to the parental MCF-7 cell line^12^. Nonetheless, even though questions have been raised about the reproducibility of results with MCF-7 cells^13^, many laboratories assume that by using cells obtained from a cell bank, standardizing protocols, limiting the number of passages, and employing SNP or STR cell authentication techniques would ensure that “their sub-clone” will behave with sufficient stability and reproducibility.

Our experience is that this may not be necessarily sufficient. Based on data from our Human Toxome Project^14^,^15^, we demonstrate by various techniques that there can be marked cellular and phenotypic heterogeneity in a single batch of cells from a cell bank that are invisible with the usual STR cell authentication protocols, and that this heterogeneity has serious consequences for reproducibility and primary outcomes of experiments.

## Results

As part of our “Mapping the Human Toxome” project, two laboratories (Brown University [BU] and Johns Hopkins University [JHU]) used MCF-7 cells from the same ATCC lot (lot number 59388743, passage 147) combined with strict adherence to standards for validation (standard operations protocols, formal training, and transfer) for cell culture and analytic methods^14^ including the recommendations for Good Cell Culture Practice^16^.

**Figure 1:**
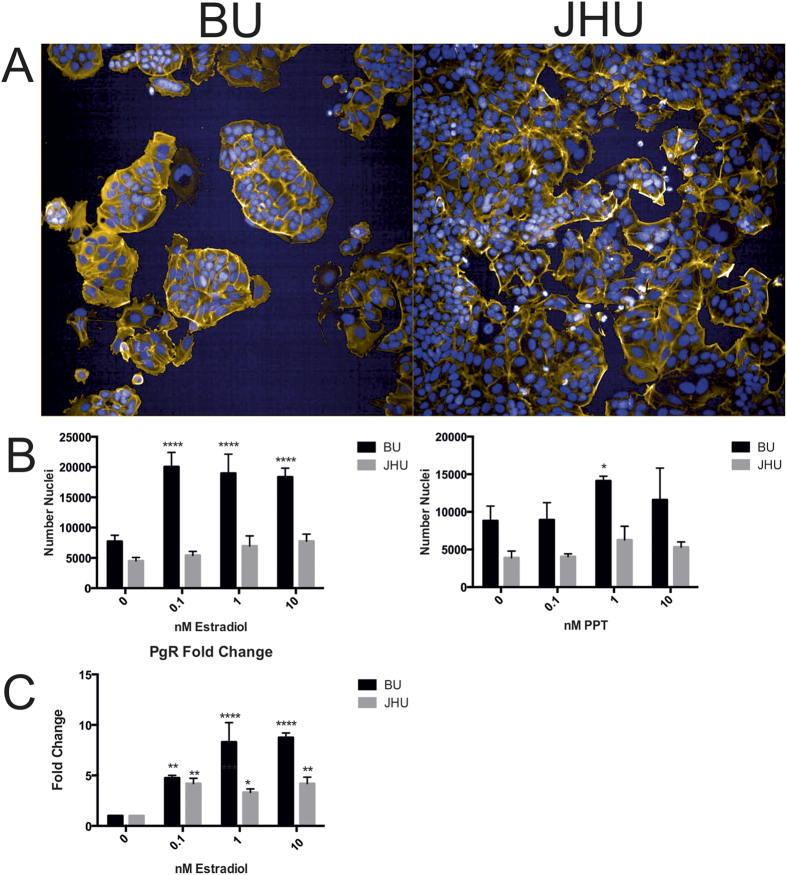
Morphological, phenotypical and gene expression differences of MCF-7 cells from the same ATCC batch (passsage number 154 JHU & passage number 150 BU). (**A**) MCF-7 sublines (JHU & BU) display distinct morphological differences. At 0 hours, BU MCF-7 cells and JHU cells have unique morphologies. MCF-7 cells grown and expanded at BU grow in large aggregations while JHU cells grown in the BU laboratory are flat, with cobblestone morphology. (**B**) Following 72 hours of exposure to estradiol (E2), BU MCF-7 cells displayed significant increases in proliferation (cell count) at concentrations of 0.1, 1 and 10 nM, while JHU cells did not have a significant change in cell count (left). Exposure to the estrogen receptor alpha agonist propyl pyrazole triol (PPT) for 72 hours resulted in a significant increase in proliferation at concentrations of 0.1, 1.0 and 10 nM in the BU subline, while JHU cells did not exhibit significant changes (right). (**C**) Gene expression analysis of the estrogen receptor target progesterone receptor (PgR) following 6 hours of exposure to E2 indicated that BU MCF-7 cells are responsive to low levels of estrogen. All experiments have been performed in one laboratory (BU) by one individual to exclude any possible inter-laboratory and/or inter-operator effects on reproducibility. *p < 0.05, **p < 0.01, ***p < 0.001, ****p < 0.0001.

In a first step, this work included expansion of the cells from the original ATCC vials using three passages for BU and eight passages for JHU, respectively to create vials for use in experiments, each of which were then passaged up to 10 times after which another vial was thawed for continuing experiments. Recommended genomic typing of short tandem repeat markers (STR) showed that all MCF-7 cell markers were the same lengths as provided by the reference ATCC genotyping panel for all 9 typed markers (Table 1). Nonetheless, significant differences were observed between the two laboratories in terms of phenotype, gene expression patterns, metabolomics, and (most crucially) sensitivity to estradiol-driven proliferation. To exclude any possible inter-laboratory and/or inter-operator effects, the JHU cells were shipped to BU to verify the results. The results of the morphological, phenotypical and gene expression differences that have been performed in one laboratory (BU) by one individual are given in Fig. 1: Morphologic assessment of the MCF-7 cells showed that BU cells grow in large aggregations while JHU cells grow flat, with cobblestone morphology (Fig. 1A). Following 72 hours of exposure to estradiol (E2), BU MCF-7 cells displayed significant increases in proliferation (cell count) at concentrations of 0.1, 1 and 10 nM, while JHU cells did not have a significant change in cell count (Fig. 1B, left). Exposure to the estrogen receptor alpha agonist propyl pyrazole triol (PPT) for 72 hours resulted in a significant increase in proliferation at concentrations of 0.1, 1.0 and 10 nM in the BU subline, while JHU cells did not exhibit significant changes (right). Gene expression analysis using quantitative PCR of the estrogen receptor target progesterone receptor (PgR) following 6 hours of exposure to E2 indicated that BU MCF-7 cells are responsive to low levels of estrogen (Fig. 1C). To exclude possible laboratory/technician influences, cells were exchanged between the laboratories, but the differences persisted (data not shown).

**Figure 2:**
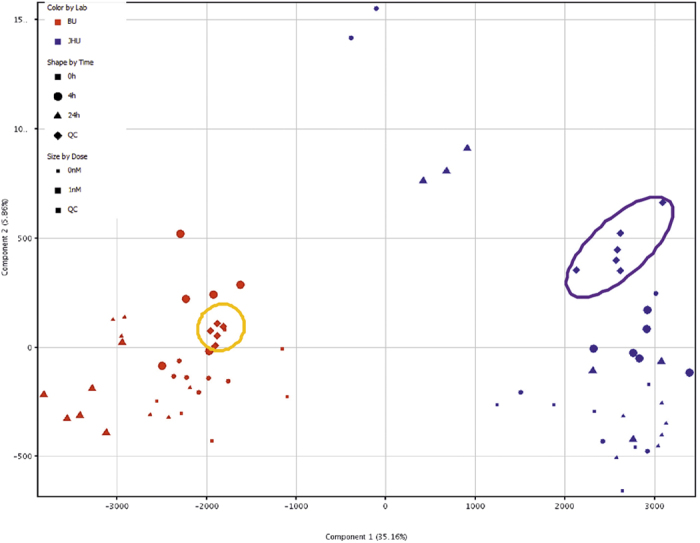
Principal Component analysis (PCA) of MCF-7 cells LC-MS metabolomics data from the same ATCC batch. MCF-7 cells were treated with 0 nM or 1 nM E2 for 4 hours or 24 hours either at Brown University (BU) or Johns Hopkins University (JHU). The data point colors in the graph represent samples from Brown University and at passage number 150 (BU, red) and Johns Hopkins University at passage number 154 (JHU, blue). The shapes represent the treatment time (square: 0 h, circle: 4 h and triangle: 24 h) while the size represents the experimental condition (small: controls and big: 1 nM E2 treatment). QC samples from each were also analyzed. They represent a pool of all samples in an individual experiment (diamond). A total of 1048 features were identified in the two experiments and were used for the multivariate analysis shown here.

**Figure 3:**
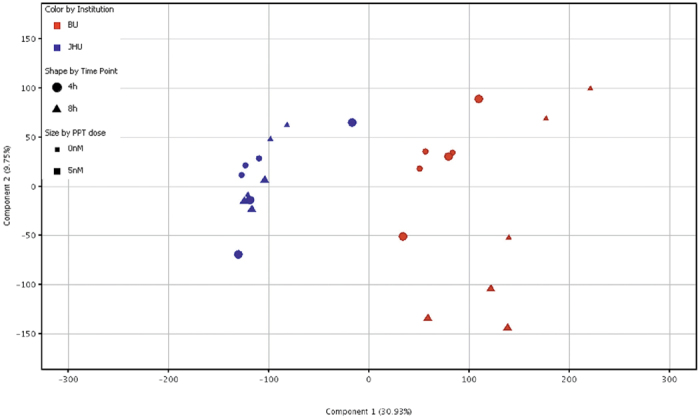
Principal Component analysis (PCA) of MCF-7 cells Gene Expression microarray data from the same ATCC batch. MCF-7 cells were treated with 0 nM or 5 nM propyl pyrazole triol (PPT) for 4 hours or 8 hours (cell culture details see Materials and Methods) either at Brown University (BU) at passage number 150 or Johns Hopkins University (JHU) at passage number 154. The colors in the graph represent samples from Brown University (BU, red) and Johns Hopkins University (JHU, blue). The shapes represent the treatment time (circle: 4 h and triangle: 8 h) while the size represents the experimental condition (small: 0 nM controls and big: 5 nM PPT treatment). A total of 29,787 entities representing detected probes were used for the multivariate analysis shown here.

**Figure 4:**
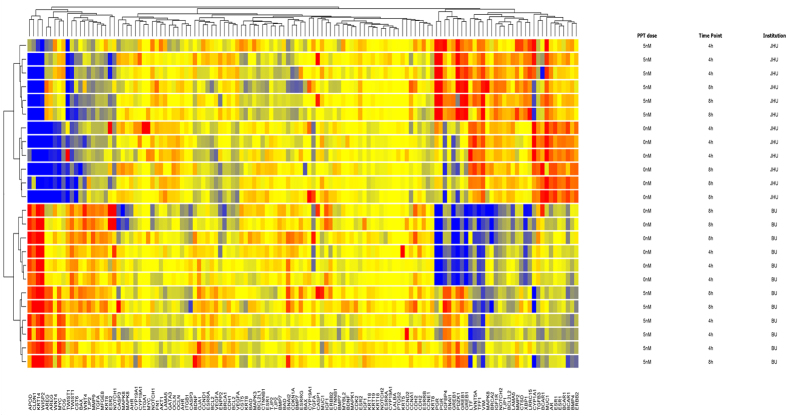
Unsupervised hierarchical clustering of MCF-7 cells Gene Expression microarray data from the same ATCC batch. MCF-7 cells were treated with 0 nM or 5 nM propyl pyrazole triol (PPT) for 4 or 8 hours (cell culture details see Materials and Methods) either at Brown University (BU) at passage number 150 or Johns Hopkins University (JHU) at passage number 154. 84 genes covering estrogen receptor signaling, breast and ductal morphogenesis, cellular growth and differentiation, proliferation, tumor progression and epithelial to mesenchymal transition have been selected from the literature^17^. Cluster algorithm used Euclidean distances and Wards linkage criteria on entities and conditions of probes encoding genes. Gene Symbols in bottom labeling columns (often multiple probes represent each gene on microarray). Label plots on the right show conditions corresponding to PPT dose (nM), Time Point (hours) and Institution (Johns Hopkins University, JHU; Brown University, BU), respectively. Color range represents data baselined to the median and log 2 transformed. Comparing two sample from 0 (yellow) to +2 (red) or −2 (blue) would be 4 fold change.

To verify whether these striking differences can be generalized, two omics technologies, i.e. gene expression microarrays and untargeted LC-MS metabolomics, have been performed as well. The experiments included different concentrations of E2 and PPT at various time points. Principal Component analysis (PCA) of the obtained 1048 features from the untargeted metabolomics experiments indicates that the two laboratories responded differentially to the E2 treatment (Fig. 2), which was more pronounced than any agonist effect. The QC samples (i.e. the pool of all samples in each experiment per lab) group together for each experiment but are well separated in the PCA. This demonstrates good technical reproducibility, while a clear distinction between the two sublines is observed. Moreover, all experimental conditions from an individual experiment group together, but separately from the other subline. Very similar effects can be seen in the PCA of the gene expression microarray data which indicate that the transcriptional state of the cells is already different in the control untreated cells and this difference persists in treated samples (Fig. 3). This can also be seen in a heat map and unsupervised hierarchical clustering from the same experiment of 84 genes selected from the literature^17^ covering estrogen receptor signaling, breast and ductal morphogenesis, cellular growth and differentiation, proliferation, tumor progression and epithelial to mesenchymal transition (Fig. 4).

**Figure 5:**
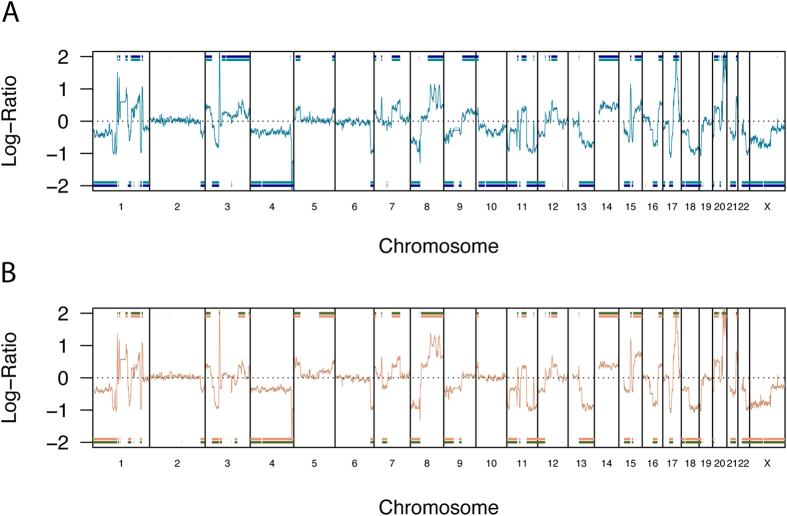
Reproducibility of CGH for two technical replicates of MCF-7 cells. Comparative Genomic Hybridization of two technical replicates of MCF-7 cells from ATCC lot number 59388743 from Johns Hopkins University at passage number 154 (JHU P154, (**A**) darkblue and darkcyan), and Brown University and at passage number 150 (BU P150, (**B**) darkolivegreen and darksalmon) versus human female reference DNA. Within (**A,B**) respectively, only very minor differences can be seen showing very good reproducibility of CGH. Significant genomic differences detected by the Aberration Detection Method 2 (ADM-2) are indicated by the respective horizontal lines at −2 and +2, respectively. All experiments have been performed in one laboratory (JHU) by one individual to exclude any possible inter-laboratory and/or inter-operator effects on reproducibility.

**Figure 6:**
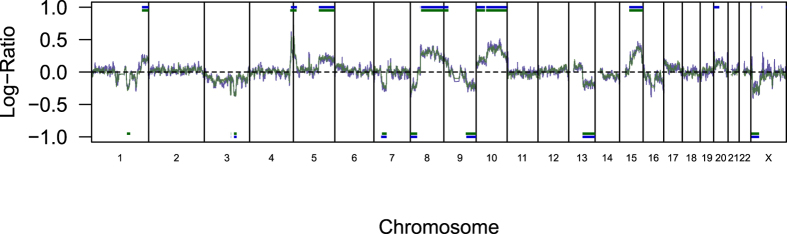
Direct CGH of MCF-7 genomics DNA from two original ATCC vials of the same batch and after short culture. Direct Comparative Genomic Hybridization (CGH) of MCF-7 genomic DNA derived from ATCC lot number 59388743, passage 147. Genomic DNA has been directly prepared from two original ATCC vials with the same lot number but shipped at two different time points to two different laboratories (blue) and after few passages in cell culture in the related laboratories (green; JHU passage number 154, BU passage number 150). For the comparative analysis the JHU samples have been defined as reference samples. Both CGH show significant genomic differences on chromosomes 1, 3, 4, 5, 7, 8, 9, 10, 13, 15, 20 and X as detected by the Aberration Detection Method 2 (ADM-2), which are very similar in both comparisons and are indicated by the respective horizontal lines at −1 and +1, respectively. Note, that to show the smaller differences between the different MCF-7 samples in comparison to MCF-7 versus normal female genome (Fig. 5), the Y-axis has been changed. All experiments have been performed in one laboratory (JHU) by one individual to exclude any possible inter-laboratory and/or inter-operator effects on reproducibility.

These results suggested that MCF-7 cells of the same batch from a cell bank include subpopulations with different genomic backgrounds, and that this could explain the phenotypic differences. To explore this possibility, we looked for possible genomic differences using genome-wide Comparative Genomic Hybridization (CGH) in the two MCF-7 subpopulations. We also included genomic DNA directly prepared from second untouched never thawed ATCC vials with the same lot number that had been continuously stored since 2011 in liquid nitrogen at both laboratories (ATCC BU and ATCC JHU). Technical replicates of MCF-7 genomic DNA from MCF-7 cells being used for experiments after a few passages hybridized against human Caucasian female reference DNA showed only small differences of short length, which are simply expected by statistical chance because of the >100,000 involved CGH probes (Fig. 5). However, the direct comparative hybridization of DNA from genomic DNA directly prepared from two original ATCC vials and the cell cultures derived from them provided surprising results: MCF-7 is not just a mixture of cells with heterogeneous genetic backgrounds from the same donor so they will have similar STR patterns, - in fact, almost the same significant genomic differences on chromosomes 1, 3, 4, 5, 7, 8, 9, 10, 13, 15, 20 and X as seen after a few passages in cell culture can already be detected from genomic DNA directly prepared from the two original ATCC vials from the same lot number (Fig. 6). This observation indicates that the original vials from the same ATCC lot already showed most of the genetic differences, and the genetic heterogeneity introduced by passaging at the two laboratories was minimal.

## Discussion

As our data demonstrate, seemingly similar cells from the same ATCC batch, that show the same STR genotypes for cell line authentication and applied current recommendations on Good Cell Culture Practice, produced starkly different results for key outcomes – proliferation, expression of estrogen responsive genes, estrogen dose-response curve, metabolomics and transcriptomics. Geno- or karyotyping profiling just fulfills the purpose of authentication testing, which is to detect significant cross-contamination and cell line misidentification as defined by The International Cell Line Authentication Committee (ICLAC): “The aim of authentication is to confirm or verify the identity of a cell line, ensuring that it is derived from the correct species and donor”^18^ ^(page 4)^. In other words, the current standard ways that are used to “ensure” cell authentication do not preclude the possibility of genetic heterogeneity at a level that could fundamentally compromise reproducibility within and between laboratories and heterogeneity needs to be examined using additional characterization methods. Even simple outcomes, such as estradiol dose-response, can be divergent between two laboratories using what appear to be the “same” cells and the same protocol. Similar results for MCF-7 have already been demonstrated in the past by other studies e.g. refs 9 and 19, however, none of the reported studies showed that significant genomic differences can already be present within the same lot from a cell bank. Since the genomic DNA was directly prepared from the original ATCC vials by one operator at the same time point possible thawing, cell-culture, inter-laboratory and/or inter-operator effects can be excluded. Given the widespread use of cancer-derived cell lines not only for basic science but also for drug development and regulatory decision-taking, ensuring that such cells are adequately standardized represents a challenge going forward. These findings underscore the need for additional quality assurance in Good Cell Culture Practice and cell characterization, especially using CGH or deep sequencing, to reveal possible genomic heterogeneity and drifts within cell lines, which are not detected by geno- or karyotyping and other current recommendations. CGH compared to deep sequencing offers advantages as to costs and bioinformatics efforts needed to analyze such differences.

In this respect, MCF-7 are likely not unique. For example, studies with T47D - the second most commonly used cell line as an *in vitro* breast cancer model - as well as others^20^ suggest similar genotypic and phenotypic heterogeneity^21^,^22^,^23^. However, these have not been shown for cells from the same batch without further cultivation. Cell model heterogeneity could be an advantage under some circumstances, and MCF-7 has been proposed as “an interesting model for genetic evolution of breast tumors”^10^. But such heterogeneity can most certainly be a curse when it comes to other common applications of such cells, as it not only weakens “the direct relevance of such cultures as models of human cancer”^8^ but also “makes inter-laboratory reproduction of experimental findings difficult”^24^. It is noteworthy that, in work parallel to our own, the MCF-7 estrogen disruptor assay failed international validation by the US National Toxicology Program Interagency Center for the Evaluation of Alternative Toxicological Methods (NICEATM), mainly because of concerns about inter-laboratory reproducibility^25^. These issues are of profound concern when an *in vitro* cell model is used for investigations designed to inform patient care or hazard assessment. The combination of omics technologies does not overcome the limitations of tumor cell lines but makes their problems more evident.

## Materials and Methods

### Experimental Design

**Figure 7:**
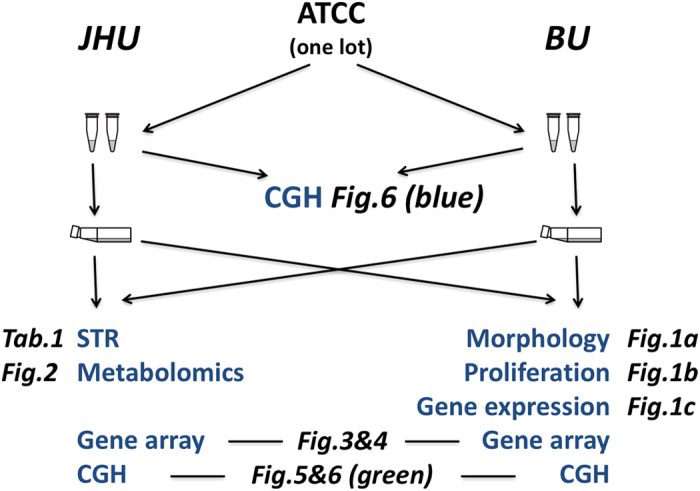


A schematic overview of the whole process from cell acquisition to experiments performed is given in Fig. 7.

#### Cell culture

Two vials of MCF-7 cells of identical lot numbers were purchased by each laboratory from the American Type Culture Collection (ATCC, Manassas, VA, USA no. HTB-22, lot number 59388743, passage 147, shipped at different time points in October and November 2011) and cells from one vial were grown using identical protocols in two labs, Brown University (BU) and Johns Hopkins University (JHU), while the other vial was never thawed. There were no differences observed in transport of source vials or handling of vials on arrival. Following thawing of one ATCC vial per laboratory the cell viability was assessed after one passage, and viability was comparable between both laboratories and with previous reports. MCF-7 cells were expanded for several passages in each lab to provide sufficient stock and experiments were performed from these stocks on passage number 3 from BU and passage 8 from JHU.

From each laboratory, one sample of MCF-7 stock was tested for contamination by forty Mollicutes species, (i.e. Mycoplasma) using GRCF’s mycoplasma test that uses a PCR based MycoDtect™ kit from Greiner Bio-One North America, Inc. (Monroe, NC) to PCR amplify the 16S–23S intergenic spacer region with a highly conserved fluorescent primer pair. Briefly, DNA is extracted from the cell culture cells and supernatant using a DNeasy Blood & Tissue kit automated on a QIAcube (Qiagen). A MycoDtect internal control is added to each sample prior to isolation to monitor the DNA extraction process and template use for the amplification. A PCR control within the PCR MasterMix allows for validation of the PCR. The labeled products are hybridized to complementary sequences on the MycoDtect chip. For each mycoplasma species, the universal probe, and internal and PCR controls are detected by five measuring points on the chip. Nonspecifically bound probes are removed by washing. The bound and labeled probes are detected by stimulation with monochromatic light and analyzed using CheckReport software.

MCF-7 cells were maintained in complete growth medium composed of DMEM-F12 (GIBCO, Life Technologies, Grand Island, NY, USA, no. 11309) supplemented with 10% fetal bovine serum (Atlanta Biologicals, Norcross, GA, USA, no. S11150), 1× nonessential amino acids (GIBCO, Life Technologies, no. 11140), 10 μg/mL bovine insulin (Akron Biotech, Boca Raton, FL, USA, no. AK8213) and 0.01 mg/ml gentamicin (Invitrogen, Life Technologies, no. 15710) in Bisphenol-A-free culture flasks. Cultures were fed every 2–3 days and passaged when 70–80% confluent. Serum used in both labs was purchased from the same lot. To control for genetic drift, MCF-7 cells from the initial thawed vial were only used in experiments for up to 10 passages.

#### Treatment of Cultures

For the RNA and metabolomics studies, the MCF-7 cells were seeded at a density of 300,000 cells/well in 6-well plates and allowed to grow for 72 hours in complete growth media. After 72 hours, cells were rinsed with 1× PBS and placed in treatment media composed of DMEM-F12 supplemented with 5% dextran charcoal stripped fetal bovine serum (DCC, Gemini Bio-products, Sacramento, CA, US, no. 100–119), 6 ng/mL bovine insulin and the same additions of nonessential amino acids and gentamicin as for maintenance culture for 48 hours. Cells were then exposed to propyl pyrazole triol (PPT, Tocris, Minneapolis, MN, USA, no 1426), 17β-estradiol (E2, Sigma Aldrich, St. Louis, MO, USA, no. E8875) or vehicle control dimethylsulfoxide (DMSO, Sigma Aldrich, no. D8418) in fresh treatment media for 2, 4, 8, or 24 hours.

#### Morphologic Assessment

MCF-7 cells from JHU and BU were seeded into a 96-well CellCarrier Optical Imaging Plate (Perkin Elmer, Waltham, MA USA, no. 6005558) at a density of 10,000 cells/well for 0 hour time point, and plated at 5,000 cells/well for the 72 hour time point. Cells were allowed to grow in 10% complete medium for 72 hours, and then placed in 5% DCC treatment media for 48 hours. Cells were then exposed to propyl pyrazole triol (PPT, Tocris, Minneapolis, MN, USA, no. 1426), 17β-estradiol or vehicle control DMSO for 24, 48 or 72 hours. Cells were fixed in formalin, washed with 1× PBS and permeabilized with 0.25% Triton X-100 for 15 minutes. Samples were stained for 20 minutes using rhodamine phalloidin (Molecular Probes, Life Technologies, no. R415, 1:2000 dilution) and DAPI (Molecular Probes, Life Technologies, no. D1306, 1:2000 dilution). Cells were imaged using the 20× water immersion objective on an Opera Phenix High-Content Imaging System (Perkin Elmer). Morphologic analysis was performed using Harmony Software (V 4.1, Perkin Elmer) to assess nuclear count, cell area, cell roundness, nuclear area and nuclear roundness. Border cells were excluded. Data was exported into GraphPad Prism (v5.01, GraphPad Software) and two-way analysis of variance and post-hoc test was performed.

#### RNA Isolation and Quantitative Real-Time PCR

Cells were scraped into TRI Reagent (Sigma Aldrich, no. T9424) and stored at −80 °C until RNA isolation and qPCR analysis. RNA was isolated using the RNEasy Mini Kit (Qiagen) per manufacturer’s instructions, and cDNA was made using the Superscript II First Strand Synthesis System per manufacturer’s instructions. qRT-PCR was performed using primers for progesterone receptor (PGR) and ribosomal protein, large P0 (RPLP0) described previously^17^. Changes in mRNA were determined using the ΔΔ-C_T_ method, data was plotted in GraphPad Prism Software and two-way ANOVA used to determine statistical significance.

#### RNA isolation and microarray experimental design

Total RNA from MCF7 cells was extracted using TRizol Reagent according to manufacturer’s instruction, and purified using RNeasy Mini Kit (Qiagen). Purified RNA was quantified by using NanoDrop ND-1000 spectrophotometer (Thermo Scientific) and the quality of RNA was assessed by using Agilent Bioanalyzer (Agilent).

100 ng of total RNA from treated and control cells were converted into cDNA and then into labeled cRNA using Agilent LowInput QuickAmp Labeling Kit (Agilent). The resulting cRNA was labeled with Cy3. Labeled cRNAs were then purified, and RNA concentration and dye incorporation were measured using NanoDrop ND-1000 spectrophotometer. Hybridization to Agilent SurePrint G3 human whole genome 8 × 60 K microarray (Agilent) was conducted following manufacture’s protocol. Microarrays were scanned with an Agilent DNA microarray scanner. Feature Extraction (11.5.1.1 version, Agilent) was used to calculate the signal intensity and ratios. After deleting non-detected probes and quantile-normalization, Principal Component Analyses have been performed with activated mean centering and scaling option (GeneSpring V13.1, Agilent).

#### Untargeted metabolomics analysis

The cell culture media was removed by gentle vacuum suction and the cells were washed two times with 1 mL of pre-warmed PBS. Any residue of PBS was removed from the wells. A solution of 700 uL dry-ice cold 80:20 (v/v) methanol/water was immediately added, and the cells were scraped and collected in a 1.5 ml Eppendorf tube. The wells were washed again with an additional 700 uL solution of methanol/water and this solution was combined with the previous one. The solution was vortexed for 1 min and then stored at −80 °C for 2 h to allow for protein precipitation. For metabolite extraction, tubes were placed on dry ice for 15 min and centrifuged at 14 000 × *g* for 5 min at 4 °C. The supernatant was transferred to a new 1.5 ml tube and placed on dry ice. Then, 300 *μ*l of 80:20 methanol/water was added to the pellet and a second extraction was performed. The combined supernatants were evaporated overnight to dryness at room temperature in a Speedvac concentrator (Savant, Thermo Fisher Scientific, Waltham, MA, USA). The dried samples were reconstituted with 60 *μ*L of 60% methanol with 0.1% formic acid and clarified by centrifugation at 14000 × *g* for 5 min. The clarified samples were transferred to HPLC vials for LC-MS measurements.

Chromatographic separations were performed using an Agilent 1260 high-performance liquid chromatography system with a well-plate autosampler (Agilent, Santa Clara, CA, USA). For aqueous normal phase (ANP) separation, a Cogent Diamond Hydride (MicroSol, Eatontown, NJ, USA) column (150 × 2.1 mm i.d., 4 *μ*m particle size, 100 Å pore size) was used for separation of metabolites. The LC parameters were as follows: autosampler temperature, 4 °C; injection volume, 5 *μ*L; column temperature, 35 °C; and flow rate, 0.4 mL/min. The solvents and optimized gradient conditions for LC were: Solvent A, 50% methanol/50% water/0.05% formic acid; Solvent B, 90% acetonitrile with 5 mM ammonium acetate; elution gradient: 0 min 100% B; 20–25 min 40% B; post-run time for equilibration, 10 min in 100% B. The LC system was coupled directly to the Q-TOF mass spectrometer. A 6520 accurate-mass Q-TOF LC-MS system (Agilent) equipped with a dual electrospray (ESI) ion source was operated in negative-ion mode for metabolic profiling. The optimized ESI Q-TOF parameters for MS experiments were: ion polarity, negative; gas temperature, 325 °C; drying gas, 10 l/min; nebulizer pressure, 45 psig; capillary voltage, 4000 V; fragmentor, 140 V; skimmer, 65 V; mass range, 70–1100 *m/z*; acquisition rate, 1.5 spectra/s; instrument state, extended dynamic range (1700 *m/z*, 2 GHz). Spectra were internally mass-calibrated in real-time by continuous infusion of a reference mass solution (standards with known mass at specific concentrations, which are introduced into the ion-source throughout the sample run to perform dynamic calibration) using an isocratic pump connected to a dual sprayer feeding into an electrospray ionization source. Data were acquired with MassHunter Acquisition software from Agilent.

For the data processing and chemometric analysis of the LC-MS untargeted data, the acquired raw data files (.d files) were first checked for quality in MassHunter Qualitative Analysis software (Agilent, version 6.0). Reproducibility of chromatograms was visually inspected by overlaying the Total Ion Chromatograms (TICs) of all samples. Data files that exhibit outlier peaks, i.e. replicates with very dissimilar chromatograms, were excluded for further processing. The raw data files were then converted to mzXML using ProteoWizard 3.0^26^. Raw LC-MS data were analyzed by the MZmine 2 software^27^ for chromatogram deconvolution, peak detection and alignment. The putative identification was achieved by online searching for the accurate *m/z* values of the peaks against HMDB and KEGG databases^28^,^29^. Those peaks were manually inspected for the quality of the EIC (extracted ion chromatograms) and also for remaining duplicate compounds names. Principal Component Analysis has been performed with GeneSpring V13.1 with activated mean centering and scaling option.

#### Comparative Genomic Hybridization

Genomic DNA was prepared using Qiagen QIAamp DNA mini Kit (#51304) following the manufacturer’s recommended procedure. 1 ug gDNA was labeled with Cy5 or Cy3 using Agilent SureTag Complete DNA labeling kit (Agilent, Part# 5190-3399) and hybridized to Agilent SurePrint G3 4 × 180 K ISCA Human CGH+SNP array (Agilent Part #G4890A, AMADID: 029830) following the manufacturer’s recommended procedure (Agilent Oliognucleotide Array-Based CGH for *“GenomicDNA Analysis: Enzymatic Labeling for Blood, Cells, or Tissues”* protocol version 7.3, Part# G4410-90010). The hybridized slides were scanned using Agilent Scanner G2505C and array data was extracted from the scanned image using Agilent Feature Extraction software version 11.5.1.1.

Data were normalized and analyzed with CytoGenomics 3.0.4.1 (64 bit) and implemented default analysis method CGH v2. Briefly, after filtering for saturated and non-uniform probes, data were normalized by GC correction with a window size of 2 kb and Diploid Peak Centralization. Aberrations were detected by the Aberration Detection Method 2 (ADM-2) with a threshold of 10 and applied mosaic aberration filter with standard build-in parameters. For the graphical visualization a moving average smoothing function of +/−50 probes has been applied and shown in the figures.

For the direct Comparative Genomic Hybridization analysis, the JHU cells have been defined as Cy3-labeled reference samples (positive log ratio defined comparative gain in BU versus JHU cells). For the indirect CGH sample, DNA was hybridized against human female reference DNA as supplied by Agilent SureTag Complete DNA labeling kit.

All CGH experiments have been performed in one laboratory (JHU) by one individual to exclude any possible inter-laboratory and/or inter-operator effects on reproducibility.

#### Short Tandem Repeat profiling

STR profiling was carried out following the ANSI/ATCC ASN-0002-2011 guidance, Authentication of Human Cell Lines: Standardization of STR Profiling. Briefly, a Promega GenePrint 10 Kit was used to polymerase chain (PCR) amplify eight short tandem repeat (STR) loci plus a gender-determining marker, Amelogenin. The PCR product was electrophoresed on an ABI Prism® 3730xl Genetic Analyzer using an Internal Lane Standard 600 (Promega). Data was analyzed using GeneMapper version 4.0 software (Applied Biosystems). Appropriate positive and negative controls were used.

All STR experiments have been performed in one laboratory (JHU) by one individual to exclude any possible inter-laboratory and/or inter-operator effects on reproducibility.

**Data and materials availability.** The CGH datasets have been deposited in the Gene Expression Omnibus (GEO) under GSE80760 (http://www.ncbi.nlm.nih.gov/geo/query/acc.cgi?acc=GSE80760). The transcriptomics microarray datasets have been deposited in the Gene Expression Omnibus (GEO) under GSE77244 (http://www.dtd.nlm.nih.gov/geo/query/acc.cgi?acc=GSE77244).

## Additional Information

**How to cite this article**: Kleensang, A. *et al.* Genetic variability in a frozen batch of MCF-7 cells invisible in routine authentication affecting cell function. *Sci. Rep.* **6**, 28994; doi: 10.1038/srep28994 (2016).

## Figures and Tables

**Table 1:** STR profiling of genomics MCF-7 DNA directly prepared from two original ATCC vials of the same ATCC batch.

Loci	ATCC BU	ATCC JHU	Brown P150	JHU P154	ATCC reference
D5S818	11,12	11,12	11,12	11,12	11,12
D13S317	11	11	11	11	11
D7S820	8,9	8,9	8,9	8,9	8,9
D16S539	11,12	11,12	11,12	11,12	11,12
vWA	14,15	14,15	14,15	14,15	14,15
TH01	6	6	6	6	6
AMEL	X	X	X	X	X
TPOX	9,12	9,12	9,12	9,12	9,12
CSF1PO	10	10	10	10	10

STR profiling of MCF-7 cells from two separate shipments but the same ATCC lot number (ATCC #HTB-22, lot number 59388743, passage number 147). ATCC BU and ATCC JHU are the STR profiles from the original shipped vials whereas BU P150 and JHU P154 are the respective STR profiles from passage 150 and 154. All four samples and the reference typing from ATCC show the same STR profiles. All experiments have been performed in one laboratory (JHU) by one individual to exclude any possible inter-laboratory and/or inter-operator effects on reproducibility.
