# Erratum: Genetic variability in a frozen batch of MCF-7 cells invisible in routine authentication affecting cell function

**DOI:** 10.1038/srep33011

**Published:** 2016-09-14

**Authors:** Andre Kleensang, Marguerite M. Vantangoli, Shelly Odwin-DaCosta, Melvin E. Andersen, Kim Boekelheide, Mounir Bouhifd, Albert J. Fornace, Heng-Hong Li, Carolina B. Livi, Samantha Madnick, Alexandra Maertens, Michael Rosenberg, James D. Yager, Liang Zhao, Thomas Hartung

Scientific Reports 6: Article number: 2899410.1038/srep28994; published online: 07 26 2016; updated: 09 14 2016

The original version of this Article contained a typographical error in the spelling of the author Liang Zhao, which was incorrectly given as Liang Zhaog. This has now been corrected in the PDF and HTML versions of the Article.

